# Primary care consultation, hospital admission, sick leave and disability pension owing to neck and low back pain: a 12-year prospective cohort study in a rural population

**DOI:** 10.1186/1471-2474-7-66

**Published:** 2006-08-14

**Authors:** Sara AC Holmberg, Anders G Thelin

**Affiliations:** 1 Research and Development Centre, Kronoberg County Council, Box 1223, SE-351 12 Växjö, Sweden; 2 Department of Public Health and Caring Sciences, Family Medicine and Clinical Epidemiology Sections, Uppsala University, Uppsala, Sweden

## Abstract

**Background:**

Neck and low back pain are common musculoskeletal complaints generating large societal costs in Western populations. In this study we evaluate the magnitude of long-term health outcomes for neck and low back pain, taking possible confounders into account.

**Method:**

A cohort of 2,351 Swedish male farmers and rural non-farmers (40–60 years old) was established in 1989. In the first survey, conducted in 1990–91, 1,782 men participated. A 12-year follow-up survey was made in 2002–03 and 1,405 men participated at both times. After exclusion of 58 individuals reporting a specific back diagnosis in 1990–91, the study cohort encompassed 1,347 men. The health outcomes primary care consultation, hospital admission, sick leave and disability pension were assessed in structured interviews in 2002–03 (survey 2). Symptoms and potential confounders were assessed at survey 1, with the exception of rating of depression and anxiety, which was assessed at survey 2. Multiple logistic regression generating odds ratios (OR) with 95% confidence intervals (95% CI) was performed to adjust the associations between reported symptoms and health outcomes for potential confounders (age, farming, workload, education, demand and control at work, body mass index, smoking, snuff use, alcohol consumption, psychiatric symptoms and specific back diagnoses during follow up).

**Results:**

Of the 836 men reporting current neck and/or low back pain at survey 1, 21% had had at least one primary care consultation for neck or low back problems, 7% had been on sick leave and 4% had disability pension owing to the condition during the 12 year follow up. Current neck and/or low back pain at survey 1 predicted primary care consultations (OR = 4.10, 95% CI 2.24–7.49) and sick leave (OR = 3.22, 95% CI 1.13–9.22) after potential confounders were considered. Lower education and more psychiatric symptoms were independently related to sick leave. Lower education and snuff use independently predicted disability pension.

**Conclusion:**

Few individuals with neck or low back pain were on sick leave or were granted a disability pension owing to neck or low back problems during 12 years of follow up. Symptoms at baseline independently predicted health outcomes. Educational level and symptoms of depression/anxiety were important modifiers.

## Background

Pain and discomfort from the neck and the low back are very common complaints in the general population. The majority of middle aged people have experienced such symptoms for shorter or longer periods [1-4]. In Sweden, musculoskeletal disorders are still the most common reason for sick leave and disability pension, although psychiatric illnesses are on the increase [5]. In spite of the fact that neck and low back pain are so frequent, have been extensively researched and carry large societal costs, we still have limited knowledge about the causes as well as the course of these conditions.

Associations between both physical workload and psychosocial factors and neck pain [6,7] and low back pain [8-10] have been reported in numerous studies. However, there is still not sufficient evidence to support causal relations regarding these factors [11]. Psychological factors seem to be important to the development of these conditions over time, to how pain is conceived and handled in terms of care seeking and sick listing [12]. There is limited data on the course of low back pain [13] and almost no data on the course of neck pain in populations over longer periods. Although the majority of pain episodes are mild, many have recurrent episodes and a minority develop persistent chronic pain [14]. How health care consumption, sick leave and disability pensions develop over a longer time span in relation to previously reported symptoms is insufficiently studied. In view of the major individual suffering and societal costs for sick leave compensation and disability pensions in Sweden and comparable countries, studies of prospective data on these matters are a pressing matter [15].

In the research project "Health through work" we have followed a cohort of initially occupationally active middle-aged farmers and rural non-farmers over 12 years. At the baseline survey in 1990–91 the farmers generally reported more musculoskeletal symptoms than non-farmers [16]. Despite this, farmers had not sought medical care more often than non-farmers and had reported less sick listing. The two groups differ considerably in physical and psychosocial background characteristics and therefore the cohort is suitable for studies on musculoskeletal health outcomes. The purpose of the present study was to describe the frequency of primary health care consultations, hospital admissions, sick leave and disability pensions due to neck and low back pain during 12 years of follow up in relation to reported symptoms at baseline taking potential confounders into account.

## Methods

### Study population

In 1989, a study cohort of 2,351 male farmers and non-farmers (aged 40–60 years), from nine different municipalities across Sweden, was created for prospective studies of primarily salutogenes. The municipalities were selected to cover known morbidity gradients across the country and to include areas with various types of farming. The farmers were identified using the Swedish Register of Farming, and occupational activity in farming (>25 hours per week) was thoroughly checked. The non-farmers were sampled from the national population register and matched to the farmers by sex, age and residential area. The non-farmers had to be occupationally active in other than farming. The sampling procedure has been described in more detail previously [16-19].

The study cohort was invited to participate in an extensive health survey in 1990–91, and 1,782 men (75.8%) participated in this baseline survey (survey 1). In 2002–03, 1,589 (67.6%) men participated in a second similar survey (survey 2). A description of the study cohort and reasons for non-participation is given in table 1. Both surveys were performed with specially trained personnel traveling to the various areas, and they were carried out as separate research projects and not part of any ordinary health program. The participation rate was somewhat higher among the farmers than among the non-farmers although the setting was the same, and the groups were not addressed differently. Fifty-eight men reporting a specific back diagnosis at survey 1 (rheumatoid arthritis, ankylosing spondylitis or disc herniation) were excluded before analyses in order to limit the follow-up analysis to those with unspecific symptoms. Hence, of the 1,405 men participating at both surveys, 1,347 constitute the study cohort.

### Outcome measures

The outcomes were primary care consultations, hospital admissions, sick leave and disability pensions owing to a neck or back diagnosis (International Classifications of Diseases, ICD-9 720–724) [20] during the follow up period of 12 years. The outcomes were assessed in a structured interview by an experienced physician at survey 2. Each outcome was dichotomized so that one or several consultations, hospital admissions or sick leaves due to neck or back problems was opposed to not having any consultation, admission or sick leave of that kind.

### Neck and low back symptoms

All studied variables except rating of psychiatric symptoms were measured at survey 1. Neck pain and low back pain ever during lifetime and during the last year were assessed in a questionnaire [16]. The neck questions posed included neck or shoulder pain/discomfort but this is denoted neck pain in this report to facilitate reading. Three groups were identified: (1) those who reported no neck or low back problems before the study period, (2) those who reported neck or low back problems previously but not during the last year prior to survey 1 (previous neck and/or low back pain) and (3) those who reported neck or low back problems previously and during the year before survey 1 (current neck and/or low back pain). Very few individuals reported neck and/or low back problems only during the last year and not before. These were included in the latter group.

### Confounders

Physical workload was assessed in a structured interview by an experienced physician in occupational medicine as the reported average number of hours working in a sitting or standing position, with a moderate, heavy or very heavy workload during an average working day according to Edholm's activity scale [21]. Educational level was measured on a five grade scale (from compulsory school to university). Experienced demands at work and perceived control in the work situation were assessed in the questionnaire according to Karasek and Theorell [22]. Weight and height were measured with standard procedures and body mass index (BMI) calculated as weight in kilograms divided by height in meters squared (kg/m^2^). Tobacco and alcohol consumption were assessed in a structured interview. Tobacco consumption was analyzed with two dichotomous variables, namely current daily smoking and current snuff use (smokeless tobacco). Average alcohol intake, computed as grams of pure alcohol consumed per week, was based on frequency of alcohol intake, type of beverage consumed and amount consumed on each occasion. Presence of psychiatric symptoms was assessed with the Hopkins Symptom Check List-25 (HSCL-25) at survey 2 [23]. The scale consists of 25 items covering common psychiatric symptoms in the area of anxiety and depression graded from 1 (not at all) to 4 (very much).

The study was approved by the Research Ethics Committee at the Karolinska Institute in Stockholm, Sweden (Dnr 90:19) and by the Regional Ethical Board, Uppsala, Sweden (Dnr 2005:107). The research was carried out in compliance with the Helsinki declaration and all participants gave their informed consent.

### Statistical analyses

The statistical analyses were conducted using SPSS^® ^version 13.0. Comparisons between groups were done using t-test for continuous data and Chi^2^-test for categorical data. Multiple logistic regressions, generating odds ratios (OR) with 95% confidence intervals (95% CI), with backward removal of covariates on the 0.10 level were performed to adjust the associations between reported symptoms at survey 1 and outcome measures for possible confounding factors. In the models, educational level was handled numerically as an index ranging from one to five. The internal non-response for various covariates was generally very low. The only exception was demands at work, where 8.8% had missing data. All participants contributed data on outcome measures and BMI. All tests were two-tailed. A p-value of 0.05 was regarded as statistically significant.

## Results

In the follow-up cohort based on those 1,347 men who participated in both surveys, 211 men (15.7%) had had at least one primary care consultation owing to neck or back pain, 25 men (1.9%) had been hospitalized for the condition, 73 men (5.4%) had been on sick leave at least once and 39 men (2.9%) had a disability pension owing to a neck or back diagnosis (Table 2). Sixty-four men (4.8%) received a specific back diagnosis during the follow-up period.

The majority, 836 men (62.9%) reported current neck and/or low back pain during the last year prior to survey 1. No neck or low back pain was reported by 269 men (20.2%) and 224 men (16.9%) reported previous neck and/or low back pain. There was some overlap since some men reported only neck pain, some only low back pain and one group reported a combination of both (Table 2).

The four outcomes were clearly overrepresented in the group reporting current neck and/or low back pain at survey 1 (Table 3). Of those 836 reporting current neck and/or low back pain at survey 1, 174 (20.8%) had had at least one primary care consultations due to the condition, 21 (2.5%) had been hospitalized, 61 (7.3%) had been on sick leave and 36 (4.3%) had been granted a disability pension owing to a neck or back diagnosis. Only 14 (5.2%) of those reporting no neck and/or low back pain at survey 1 reported primary care consultations for neck or back pain during follow up, none had been hospitalized, five had been on sick leave and one had a disability pension.

Snuff use, psychiatric symptoms and specific back diagnoses during follow up were positively associated with all four outcomes (table 4). In addition, BMI was positively associated with hospital admission, whereas educational index and control at work were negatively associated with sick leave. Age and smoking were positively associated with disability pension, whereas educational index was negatively related to disability pension.

### Adjusted analyses

Current neck and/or low back pain at survey 1 predicted primary care consultations (OR = 4.1, 95% CI 2.2–7.5) and sick leave (OR = 3.2, 95% CI 1.1–9.2) owing to neck or low back diagnoses during follow up, even after possible confounders were taken into account (Table 5). The OR for disability pension was more modified by confounders although the trends were in the same direction as for the other outcomes. Lower age and lower sense of control at work were independent predictors of primary care consultations in the final model, whereas lower education, lower control at work, and more psychiatric symptoms were independent predictors of sick leave. Lower education and snuff use independently predicted disability pensions owing to neck or low back pain diagnoses. Higher age tended to predict disability pension but this did not reach statistical significance. Naturally, specific back diagnoses during follow up were strong independent predictors for all the outcomes. Previous neck and/or low back pain at survey 1 did not predict any of the outcomes before or after multiple adjustments (not shown in tables).

## Discussion

Current neck and/or low back pain at baseline strongly predicted primary care consultations and sick leave owing to neck or low back diagnoses during 12 years of follow up in this cohort of rural middle aged men. However, in general the outcome numbers were relatively low; approximately one fifth of those reporting current neck and/or low back pain at baseline had had a primary care consultation owing to a neck or back diagnosis, and about 7% had been on sick leave and very few had been hospitalized or granted a disability pension owing to the condition. This is in line with findings from other studies showing that neck and low back pain for the majority of patients is a mild symptom/disorder [14,24]. However, although the vast majority of those reporting neck or low back symptoms do not consult health care providers, several studies indicate that many of these individuals perceive persistent or recurrent symptoms of varying severity [25,26].

Very few of the men in our study with no current or previous neck or back pain at start reported primary care consultation, hospital admission, sick leave or disability pension owing to neck or back pain during follow up, indicating that few new cases are added in middle age. The fact that relatively few individuals report only previous neck and/or low back pain might depend on two reasons. First, these disorders go with recurrent episodes and therefore those afflicted fall in the "current" group. Second, if one experiences neck and/or low back problems for a shorter period and no recurrent episodes one might be less inclined to remember and report this.

Our study has several strengths. The most important one is the population-based cohort design and the long follow-up time. The participation rate was high considering the efforts required to attend two extensive health surveys 12 years apart. We used validated questionnaires and measurements methods [21-23] when possible. Few methods were available for evaluation of physical workload in large-scale population based studies when this project was designed in the late 1980s. The Edholm scale generates a wide spread of data and large differences between for example farmers and non-farmers [27] and showed a good correlation with physical work capacity in a sub-maximal work test (unpublished observation). The outcome data was assessed through structured physician-conducted interviews. In a previous analysis we found a good correlation between interview data on hospital admission diagnoses and registered data [17]. Missing data among participators was generally very low. The breadth of the survey with a diversity of variables to consider lowers the risk for confounder bias of our results.

Our study also has limitations. The cohort was rural-based and must be interpreted as such. Significant differences in morbidity and health care utilization have been reported between urban and rural populations, but the impact of this factor is uncertain [28-30]. The cohort included many farmers, a group previously shown to have a relatively low morbidity and to seek less health care in relation to reported symptoms [16,31]. Therefore, the outcome frequencies found in our study are probably lower than what can be expected in the general population. Another limitation, which might partly account for the low numbers, is recall bias during the 12-year follow up. However, as mentioned we found good congruity between interview information on previous hospital admissions with register data in a previous study [17]. There are no primary care registers available for comparable validation. In addition, this study was restricted to men. Gender differences regarding the studied outcomes might be at hand but this cannot be evaluated from the available data. Neck and low back outcomes were not separated since the conditions were considered to indicate the same type of disorder and, owing to the limited number of cases generating outcomes. There were too few cases with hospital admissions owing to neck or low back diagnoses to allow for analyses. The calculation of ORs for disability pension was also limited by low numbers, rendering a wide confidence interval. A larger sample would probably result in this outcome being statistically significant as well.

The ORs for primary care consultation, sick leave and disability pension during follow up in relation to current symptoms at baseline were only changed to some extent when other covariates were taken into account, even though some of the covariates were independent predictors of the outcomes. Higher education significantly lowered the ORs for sick leave and disability pension, a result that is well in line with the findings of others [32]. Higher rating on the depression and anxiety scale (HSCL-25) was strongly and independently associated with sick leave. However, since the HSCL-25 was assessed at survey 2 this relation is of a cross-sectional nature. Several other studies indicate depression as a strong predictor of disabling neck and low back pain [33-36]. In a parallel study analyzing functional capacity in relation to previous neck or low back symptoms, depression and anxiety was the factor most impressively related to impaired functional physical and social capacity (Thelin and Holmberg, submitted 2006).

Work related factors such as physical workload, farming and demands experienced at work showed no significant relationship with the studied outcomes. It should be noted that the study cohort represents a group with high physical workload as compared with the general population [27]. For the research seeking evidence for causality on physical work related factors and neck and low back pain it is an interesting observation that in this group with high physical workload no significant association between physical workload and subsequent health outcomes could be identified. If heavy physical workload was of major clinical importance for long term health related outcomes due to neck and low back pain this ought to be detectable in our study. It is possible that selection mechanisms may contribute to our finding. However, in a previous study of farmers no healthy worker effect was discerned for musculoskeletal disorders or general morbidity [37]. Among lifestyle factors, smoking, alcohol consumption and BMI held no independent predictive power for any of the outcomes. However, surprisingly, snuff use (smokeless tobacco) strongly predicted disability pension owing to a neck or back diagnosis even after other confounders were considered in the final model. Very few studies exist on snuff use and musculoskeletal symptoms and disorders. A multiple adjusted analysis on infantry conscripts showed snuff use to be an independent risk factor for musculoskeletal injury during a training period [38]. Smokeless tobacco has been proposed as a risk factor for osteoporosis [39]. Our finding, snuff use as a predictor of disability pension owing to neck or back diagnoses, must be interpreted cautiously but certainly warrants further studies, especially as the prevalence of snuff use is increasing in Sweden.

## Conclusion

In this study, we have shown that neck and low back symptoms predicted primary care consultations and sick leave owing to neck or low back diagnoses during 12 years of follow up. However, relatively few of the men with symptoms generated health care utilization, sick leave or disability pensions due to the condition. Higher educational level significantly lowered the risk of sick leave and disability pension, whereas depression and anxiety significantly increased the risk of sick leave. These variables seem to be more important to consider than the traditional work related factors focused on in many clinical and rehabilitation settings.

## Competing interests

The author(s) declare that they have no competing interest.

## Authors' contributions

The two authors contributed equally to the concept of the study, acquisition of data, analysis and interpretation of data and the drafting of the manuscript.

## Pre-publication history

The pre-publication history for this paper can be accessed here:



## Figures and Tables

**Table 1:** Description of the study cohort.

	n	%
Total sample	2,351	100.0
		
Participation survey 1 (1990–91)	1,782	75.8
		
Non-participation survey 1	569	24.2
deceased	6	0.2
ill	36	1.5
unable to attend	48	2,0
unwilling to attend	157	6.7
did not attend, unknown reason	304	12.9
not retrievable	11	0.5
moved	7	0.3
		
Participation survey 2	1,589	67.6
		
Non-participation survey 2	762	32.4
deceased	143	6.1
ill	49	2.08
unable attend	82	3.49
unwilling to attend	192	8.17
did not attend, unknown reason	294	12.5
not retrievable	2	0.1
		
Participation in both surveys	1,405	59.8^1^
		
Specific back diagnosis survey 1	58	2.5
		
Study cohort for analyses	1,347	57.3

^1 ^63.6% of those still alive.

**Table 2:** Frequency of outcomes, neck and low back symptoms and potential confounders in the study cohort (N = 1,347).

	n	%	mean	sd
**Neck and low back outcomes**				
Primary care consultation	211	15.7		
Hospital admission	25	1.9		
Sick leave	73	5.4		
Disability pension	39	2.9		
				
**Neck and low back symptoms**				
No neck pain^1^	602	45.2		
Previous neck pain^2^	152	11.4		
Current neck pain^3^	579	43.2		
				
No low back pain^1^	473	35.4		
Previous low back pain^2^	278	20.8		
Current low back pain^3^	584	43.7		
				
No neck or low back pain^1^	269	20.2		
Previous neck and/or low back pain^2^	224	16.9		
Current neck and/or low back pain^3^	836	62.9		
				
**Potential confounders**				
Age, years	1,347		50.1	5.9
Farmer	794	58.9		
Workload, units	1,327		198.7	84.8
Educational index	1,318		2.2	1.3
Compulsory school	516	39.2		
Vocational school	430	32.6		
Secondary school	151	11.5		
College	96	7.3		
University	125	9.5		
Demands, units	1,227		12.8	2.7
Control, units	1,312		7.1	1.2
Body mass index, kg/m^2^	1,347		26.3	3.1
Smokers	285	21.2		
Snuff users	192	14.3		
Alcohol consumption, grams/week	1,343		24.3	28.6
Psychiatric symptoms (HSCL-25)	1,346		1.28	0.28
Specific back diagnosis during follow up	64	4.8		

^1 ^No neck or low back problems before the study period.

^2 ^Neck or low back problems previously but not during the last year prior to survey 1.

^3 ^Neck or low back problems during the year before survey 1, irrespective of previously or not.

**Table 3:** Neck and low back health outcomes during follow up according to reported symptoms at survey 1 (N = 1,347).

	Primary care		Hospital admission		Sick leave		Disability pension	
	n	%	n	%	n	%	n	%

No neck or low back pain^1 ^(n = 269)	14	5.2	0		5	1.9	1	0.4
								
Previous neck or/and low back pain^2 ^(n = 224)	22	9.8	4	1.8	7	3.1	2	0.9
								
Current neck or/and low back pain^3 ^(n = 836)	174	20.8	21	2.5	61	7.3	36	4.3

^1 ^No neck or low back problems before the study period.

^2 ^Neck or low back problems previously but not during the last year prior to survey 1.

^3 ^Neck or low back problems during the year before survey 1, irrespective of previously or not.

**Table 4:** Distribution of possible confounders according to neck and low back health outcomes (N = 1,347).

	Primary care consultation			Hospital admission			Sick leave			Disability pension		
	
	No	Yes		No	Yes		No	Yes		No	Yes	
	
	mean/%	mean/%	p^1^	mean/%	mean/%	p^1^	mean/%	mean/%	p^1^	mean/%	mean/%	p^1^
Age	50.2	49.4	0.052	50.1	50.4	0.837	50.1	50.0	0.859	**50.0**	**52.4**	**0.013**
Farmer,%	59.0	58.8	0.954	58.9	64.0	0.604	59.3	52.1	0.218	59.0	56.4	0.744
Workload	198.3	201.0	0.677	198.6	203.4	0.786	199.4	187.3	0.239	199.1	185.2	0.332
Education index	2.2	2.1	0.753	2.2	2.3	0.641	**2.2**	**1.8**	**0.036**	**2.2**	**1.6**	**0.000**
Demands	12.8	12.7	0.611	12.8	13.6	0.144	12.8	12.4	0.235	12.8	12.8	0.901
Control	7.1	7.0	0.129	7.1	7.0	0.870	**7.1**	**6.7**	**0.026**	7.1	6.7	0.058
Body mass index	26.3	26.2	0.738	**26.3**	**27.5**	**0.046**	26.4	26.3	0.811	26.3	26.8	0.332
Smokers, %	20.6	24.2	0.248	21.0	32.0	0.182	20.8	28.8	0.103	**20.7**	**38.5**	**0.007**
Snuff users, %	**13.2**	**19.9**	**0.011**	**14.0**	**28.0**	**0.047**	**13.6**	**26.0**	**0.003**	**13.8**	**30.8**	**0.003**
Alcohol consumption	23.4	27.8	0.105	24.2	32.9	0.346	24.1	28.8	0.314	24.1	32.9	0.224
Psychiatric symptoms	**1.28**	**1.32**	**0.032**	**1.28**	**1.42**	**0.013**	**1.28**	**1.38**	**0.013**	**1.28**	**1.37**	**0.049**
Specific back diagnose, %	**1.5**	**22.3**	**0.000**	**3.8**	**56.0**	**0.000**	**3.2**	**31.5**	**0.000**	**3.7**	**38.5**	**0.000**

^1 ^t-test or Chi^2^-test.

**Table 5:** Odds ratios (OR) with 95% confidence intervals (95% CI) for neck and low back health outcomes during 12 years of follow-up according to reported symptoms at survey 1 (N = 1,347).

	Primary care		Sick leave		Disability pension	
	OR	95% CI	OR	95% CI	OR	95% CI

No neck or low back pain	1		1		1	
Current neck and/or low back pain, crude	**4.79**	**2.73–8.41**	**4.16**	**1.65–10.4**	**12.1**	**1.65–88.4**
Current neck and/or low back pain, adjusted^1^	**4.10**	**2.24–7.49**	**3.22**	**1.13–9.22**	6.34	0.83–48.2
Age	**0.97**	**0.94–1.00**			1.07	1.00–1.15
Educational index			**0.75**	**0.58–0.98**	**0.47**	**0.29–0.78**
Control at work	**0.86**	**0.74–0.98**	**0.81**	**0.67–0.99**		
Snuff users					**3.46**	**1.35–8.84**
Psychiatric symptoms			**3.53**	**1.53–8.15**		
Specific back diagnosis during follow up	**18.9**	**8.9–40.1**	**11.4**	**5.43–23.7**	**26.6**	**10.4–67.9**

^1 ^Variables entered at step 1: age, farmer, workload, educational index, demands, control, body mass index, smoking, snuff use, alcohol consumption, psychiatric symtoms and specific back diagnosis during follow up.

Adjusted ORs based on multiple logistic regression with backward removal of covariates with p < 0.10.
